# Novel voriconazole-loaded hyalurosomes optimized for enhanced skin penetration and antifungal activity against *Candida albicans*

**DOI:** 10.1007/s13346-025-02007-3

**Published:** 2025-11-10

**Authors:** Amparo Nácher, José-Esteban Peris, Raquel Taléns-Visconti, Octavio Díez-Sales, Maria Letizia Manca, Maria Manconi, Iris Usach

**Affiliations:** 1 Dept. of Pharmacy and Pharmaceutical Technology and Parasitology, Faculty of Pharmacy and Food Sciences, University of Valencia, Av. Vicent Andrés Estellés s/n, Burjassot, Valencia, 46100 Spain; 2 Instituto Interuniversitario de Investigación de Reconocimiento Molecular y Desarrollo Tecnológico (IDM), Universitat Politècnica de València, Av. Vicent Andrés Estellés s/n, Burjassot, Valencia, 46100 Spain; 3 Dept. Scienze della Vita e dell’Ambiente, University of Cagliari, Via Ospedale 72, Cagliari, 09124 Italy

**Keywords:** Voriconazole, Hyalurosomes, Candida albicans, Topical administration, Antifungal activity

## Abstract

Cutaneous candidiasis, mainly caused by *Candida albicans*, is a growing global health concern and is listed by WHO as a high-priority fungal threat. Suboptimal penetration of conventional vehicles limits the efficacy of current topical antifungals, increasing the risk of severe and invasive infections. Therefore, there is an innovative research field in advanced topical delivery systems to improve drug deposition, retention and antifungal efficacy. The main objective of this work was to develop nanocarriers based on hyalurosomes for the delivery of voriconazole (VCZ) and evaluate their potential to enhance the drug’s cutaneous penetration and antifungal activity. Four VCZ-loaded hyalurosomal formulations were prepared (H1-H4) by modulating the proportions of phospholipid and polyols. Although changes in some physicochemical properties were observed, all the VCZ-loaded nanosystems were nanosized (< 140 nm), spherical, multilamellar and exhibited high entrapments efficiencies (> 72%), excellent biocompatibility with human keratinocytes and potent antifungal activity against *C. albicans*. VCZ release from formulation H1 (1% phospholipid, 10% ethanol) followed a Fickian mechanism, while H2–H4 (4–10% phospholipid, 2.5–10% ethanol) exhibited anomalous diffusion involving both diffusion and matrix relaxation or erosion. Additionally, H1 and H2 (1–4% of phospholipid, 10% ethanol) achieved significantly enhanced drug penetration into deeper skin layers and superior in vivo antifungal efficacy compared to VCZ dispersion. The results highlight the potential of hyalurosomes as a next-generation topical antifungal delivery system, effective against both superficial and invasive candidiasis, with formulations H1 and H2 emerging as the most promising candidates for the treatment of the more invasive forms.

**Graphical abstract:**

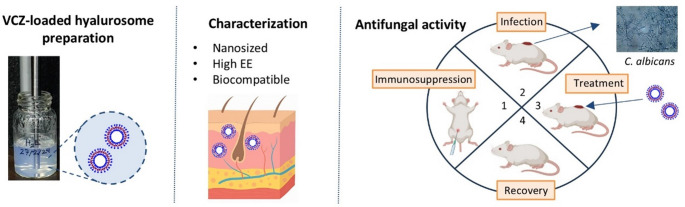

## Introduction

Skin fungal infections, or dermatomycoses, rank among the most prevalent infectious diseases, with estimates suggesting that more than a quarter of the world’s population will experience a dermatomycosis at some stage in their lifetime [1]. Recent trends indicate a rising incidence and severity of these conditions, primarily driven by risk factors such as the widespread use of broad-spectrum antibiotics, antineoplastic agents and immunosuppressive drugs [2]. This includes paediatric patients with leukaemia, who are at increased risk of developing chronic disseminated candidiasis [3]. Importantly, these infections are not limited to immunocompromised patients as fully healthy individuals, including athletes and gym users, are also frequently affected, underscoring the substantial clinical burden and propensity for recurrence that dermatomycoses generate [2], thus posing a significant clinical challenge. In this context, candidiasis is a common fungal infection mostly caused by yeasts of the *Candida species*, mainly *Candida albicans*. In fact, it was classified as one of the four highest priority fungi for global public health in the first-ever fungal priority pathogens list published by WHO in 2022 [4]. Candidiasis can affect various parts of the body, ranging from superficial mucocutaneous infections to invasive systemic disease, which may involve multiple organ systems and become life-threatening, particularly in vulnerable patients [5, 6]. In this sense, in April 2025, WHO also published its first-ever reports on fungal tests and treatments, showing the urgent need for innovative research and development [7].

The most widely used antifungal agents are included in the class of azoles, especially imidazoles. Voriconazole (VCZ) a second-generation triazole agent approved in 2002 [8], specifically inhibits the enzyme 14α-demethylase, essential for the synthesis of ergosterol, a component of the fungal cell membrane. By blocking ergosterol production, VCZ destabilizes the cell membrane and leads to fungal cell death [9]. However, VCZ, as many azoles, is poorly water soluble, which limits its bioavailability and antifungal effect. Formulation and drug delivery strategies could improve water-based hydration and dissolution properties, thus increasing its pharmacokinetics and a sustained release will prolong the retention of a high concentration of the azole localized at the infection site, therefore enhancing its bioavailability and therapeutic efficacy [10].

For cutaneous candidiasis, topical dosage forms are preferred, mainly due to site-specific drug delivery. The problem with conventional marketed formulations, based on gels, powders, creams, solutions or foams of imidazoles, is that high and repeated doses have to be administered, which may result in local or even systemic toxicity reducing patient adherence [11]. Moreover, their efficacy is limited in treating more invasive fungal infections [12]. Additionally, the increasing prevalence of drug-resistant strains has further compromised the efficacy of currently available formulations [9]. Consequently, the risks of more invasive and deadly infections among the general population have also increased. Hence, the need to develop new topical delivery systems for new-generation, broad-spectrum antifungals, such as VCZ, to overcome biopharmaceutical challenges associated with conventional drug delivery systems like poor retention and low bioavailability.

In this context, nanocarrier systems are gaining relevance due to their ability to enhance the diffusion of active ingredients across the skin barrier [10–12]. Among them, we demonstrated that nanostructured lipid carriers (NLCs) loaded with VCZ showed biocompatibility with human keratinocytes and improved the penetration and distribution of VCZ into deeper skin layers, offering superior antifungal activity [13]. Therefore, the vehicle composition of a topical delivery system may significantly affect drug release and skin penetration, thereby affecting biological activity. In this sense, liposomes have been successfully used to improve the topical efficacy of some drugs, proteins and natural ingredients by modifying their local penetration capacity into the skin and their biodistribution [14–16]. Hyalurosomes are a special type of liposomes characterized by the coupling of phospholipids with sodium hyaluronate, resulting in vesicles with greater size and mechanical stability owing to the immobilizing effect of the hyaluronan polymer. These vesicles are typically prepared by integrating sodium hyaluronate directly into the lipid bilayer or dispersion medium using methods such as thin-film hydration, ethanol injection, and sonication. These techniques yield vesicles of controlled size and high encapsulation efficiency (EE), with hyaluronic acid (HA) actively participating as a stabilizing, hydrating and functionalizing agent. Experimental evidence demonstrates the clinical utility and therapeutic potential of hyalurosomes. For example, curcumin-loaded hyalurosomes have exhibited marked antioxidant and anti-inflammatory effects that support re-epithelialization in vivo, while hyalurosomes loaded with liquorice root extract, oleuropein, lentisk oil or lavender extract have shown superior modulation of skin inflammation, oxidative stress and wound healing compared to conventional delivery systems [17–19]. Moreover, HA itself also intervenes by improving the epithelial barrier and enhancing the innate immune response, which could protect the skin from microbial infections. In fact, HA has been shown to possess antimicrobial activity against *Candida* [20]. In addition, hyalurosomes have also been explored as functionalized carriers for cancer therapy; for example, genistein-PEGylated hyaluophytosomes and Gen-hyaluophytosomes were developed for breast cancer treatment, as reported by Komeil et al. [21]. Taken together, these studies highlight the versatility and therapeutic potential of hyalurosomes.

The aim of this study is the design and characterization of novel nanosystems for voriconazole delivery enriched with HA, thus offering a novel strategy for the topical management of candidiasis. If successful, our approach could not only increase the efficacy of VCZ but also address current challenges such as poor retention, low bioavailability and the emergence of drug resistance, ultimately reducing systemic complications that could lead to hospitalizations and associated deaths.

## Materials and methods

### Reactants

The following compounds were purchased from Sigma-Aldrich (Madrid, Spain): dimethyl sulfoxide (DMSO), RPMI 1940 and 3-(4,5-dimethylthiazol-2-yl)-2,5-diphenyltetrazolium bromide (MTT) tetrazolium salt. Polysorbate 80 was obtained from Guinama (Valencia, Spain) and ethanol was purchased from PanReac Applichem (Barcelona, Spain). VCZ and hyaluronic acid (HA) (supplied as sodium hyaluronate, molecular weight 200–400 KDa) were provided by Glentham Life Sciences (Corshman, UK). Sabouraud dextrose agar (SDA) and acetonitrile (AcN) were obtained from VWR chemicals (Barcelona, ​​Spain). 4-Morpholinepropanesulfonic acid (MOPS) came from Alfa Aesar (Kandel, Germany) and Eugon LT 100 from BIOKAR Diagnostics (Beauvais, France). Glucose and cyclophosphamide monohydrate were obtained from Thermo scientific (Madrid, Spain). Phospholipon^®^ 90 G was provided by Lipoid GmbH (Ludwigshafen, Germany) and glycerol from Acofarma (Madrid, Spain). Cell medium, fetal bovine serum (FBS), penicillin, streptomycin, fungizone and all the other reagents for cell studies, unless otherwise specified, were purchased from Gibco (Paisley, UK).

### Analytical method

VCZ was quantified by means of a high-performance liquid chromatography assay (HPLC) with ultraviolet (UV) detection at 255 nm, as described previously by our research group [13]. A Waters “Nova-Pack” C_18_ analytical column (4 μm, 3.9 mm × 150 mm) was used, and the mobile phase consisted of a mixture of acetonitrile, water and 0.6% trietylamine, pH 6 (35/65, v/v). The injection volume was 25 µL, and the flow rate was 1 mL/min. The HPLC equipment consisted of a quaternary pump SpectraSYSTEM P4000, an autosampler SpectraSYSTEM AS3000 and a spectrophotometric detector SpectraSYSTEM UV 6000LP. Data were processed through “Chromquest Chromatography Workstation Software Version 1.63”.

### Preparation of hyalurosomes

Four VCZ-loaded hyalurosome formulations (H1–H4) were developed and selected based on their promising physicochemical characteristics. The main differences among them were the phospholipid concentration and the composition of the hydrating solvent mixtures (Table 1).

**Table 1:** Composition of VCZ-loaded hyalurosomes

Component (%)	Formulation			
	H1	H2	H3	H4
Sodium hyaluronate	0.1	0.1	0.1	0.1
Phospholipon^®^ 90G	1	4	10	4
Polysorbate 80	0.5	0.5	0.5	0.5
Glycerin	-	-	-	7.5
Ethanol	10	10	10	2.5
Water	90	90	90	90
Voriconazole	0.05	0.05	0.05	0.05

The components of each formulation were weighed in a glass vial and mixed under magnetic stirring at 200 rpm with ethanol / 0.1% HA in water (10:90, v/v) for H1, H2, and H3, or glycerol / ethanol / 0.1% HA in water (7.5:2.5:90, v/v/v) for H4. The dispersions were left to hydrate overnight to promote phospholipid swelling and the self-assembly of phospholipid molecules into stable vesicular structures. During hydration, the phospholipids interacted with the aqueous dispersion containing HA and the lipophilic active compounds, leading to the spontaneous formation of lamellar vesicles. As a result, well-defined nanovesicles with optimal EE, particle size distribution and surface characteristics were obtained [19]. Then formulations were sonicated with 3 cycles of 5 minutes (5 s on and 2 s off, with a probe amplitude of 60%, 45% and 60%, respectively, allowing the sample cooling between each sonication) with an ultrasonic disintegrator (CY-500, Optic Ivymen system, Barcelona, Spain) to homogenize the preparation. The probe tip (CL-18 model) had a diameter of 1/8’’ and was immersed to a depth of approx. 2.5 cm in the dispersions. Sonication was performed while the vials were immersed in a water bath maintained at room temperature (~ 25 °C) to limit overheating of the mixture. At the end of the process, the formulations reached approximately 52 °C. Finally, they were sterilized by filtration (CA syringe filter; cellulose acetate; 0.22 μm). The final concentration of VCZ in hyalurosomes was 0.5 mg/ml. Empty hyalurosomes (vehicle controls) were also prepared and used as controls in physicochemical characterization, cytotoxicity assays and antimicrobial testing.

### Characterization of hyalurosomes

Transmission electron microscopy (TEM) was used to confirm vesicle formation and evaluate morphology. Samples were diluted (1:10, v/v) with distilled water, deposited onto carbon-coated copper grids (300 mesh) and stained with 2% phosphotungstic acid aqueous solution. The grids were examined under a JEM-1010 (Jeol Europe, Paris, France) transmission electron microscope equipped with a digital camera AMT RX80 and the AmtV602 software, version 602.579 at an accelerating voltage of 80 kV and magnifications ranging from 100,000x to 150,000x.

Photon correlation spectroscopy was used to analyze the mean diameter (MD) and polydispersity index (PI) using a Zetasizer Nano (Malvern Instruments, Worcestershire, UK) at 25 °C with a backscattering angle of 173 °. The same equipment was also used to measure the zeta potential (ZP) by means of the M3-PALS (Phase Analysis Light Scattering) technique, which measures particle electrophoretic mobility.

The MD, PI and ZP were monitored over 3 months of storage at 4 ± 1 °C to evaluate the stability of the formulations.

To determine the entrapment efficiency (EE), the procedure previously described by our research group was followed [22]. Firstly, the total concentration of VCZ in the hyalurosome suspension was determined by HPLC and drug recovery (DR %) was calculated according to the following equation:

1 $$DR(\% ) = \frac{{Total\:conc.\:\:in\:hyalurosome\:suspension}}{{Theoretical\:conc.}} \times \:100$$

where the theoretical concentration was 0.5 mg/mL.

The percentage of VCZ actually encapsulated in hyalurosomes was determined with the following experiment: 1 mL of VCZ-loaded hyalurosomes was introduced into the dialysis tube (Spectra/Por^®^ membranes, 12–14 kDa MW cut-off, 3 nm pore size; Spectrum Laboratories Inc., DG Breda, the Netherlands) and kept at room temperature (25 ± 1 °C) in 100 mL of distilled water under continuous stirring for 10 h. After reaching the dialysis equilibrium, a sample of 0.5 mL of the exterior aqueous medium was taken and added to 0.5 mL of AcN. The mixture was injected into the HPLC to determine the VCZ concentration in the external aqueous medium, which is assumed to be equal to the free (unencapsulated) VCZ concentration in the hyalurosomes. Additionally, the total VCZ concentration inside the dialysis tube was quantified by HPLC after disrupting the vesicles with AcN (1/10). The encapsulated drug (ED %) was calculated with the following equation:

1 $$\begin{array}{*{20}{l}}{\:ED(\% ) = \frac{{Conc.\:\:inside\:the\:hyalurosomes}}{{Total\:conc.}}}\\{ = \frac{{Total\:conc.\:\:inside\:dyalisis\:tube\: - Free\:conc.}}{{Total\:conc.\:\:inside\:dyalisis\:tube}} \times \:100}\end{array}$$

Entrapment efficiency (EE %) was obtained as follows:

2 $$\:EE(\% ) = \frac{{Conc.\:\:inside\:the\:hyalurosomes}}{{Theoretical\:conc.}} = \:\frac{{DR\:\left( \% \right)\: \times \:\:ED\:\left( \% \right)}}{{100}}$$

### In vitro release study

The release studies of the hyalurosomes were conducted using the dialysis membrane method. 1 mL of VCZ-loaded hyalurosomes was loaded into a Spectra/Por^®^ 2 standard regenerated cellulose dialysis tube with an MWCO of 12–14 kDa and clipped by standard closures. The dialysis tube was immersed into 100 mL of distilled water with a magnetic stirrer stirring at 300 rpm. At 0, 1, 2, 3, 4, 6, 8 and 10 h, 0.5 mL of the medium were removed and replaced with an equal volume of distilled water. VCZ released amounts were determined by HPLC. All experiments were performed four times.

To determine the release kinetics of VCZ from hyalurosomes, different commonly used mathematical models including zero order, first order, Higuchi, Korsmeyer–Peppas and Peppas–Sahlin were used. The equations that represent each drug release model are summarized in Table 2.

**Table 2:** Equations of the models used for fitting drug release data

Model	Equation
Zero order	$\:{Q}_{t}/{Q}_{\infty\:}\left(\%\right)={K}_{0}\bullet\:t$
First order	$\:{Q}_{t}/{Q}_{\infty\:}\left(\%\right)=100\bullet\:(1-{e}^{-{K}_{1}\cdot t})$
Higuchi	$\:{Q}_{t}/{Q}_{\infty\:}\left(\%\right)={K}_{H}\bullet\:\surd\:t$
Korsmeyer-Peppas	$\:{Q}_{t}/{Q}_{\infty\:}\left(\%\right)={K}_{K-P}\bullet\:{t}^{n}$
Peppas-Sahlin	$\:{Q}_{t}/{Q}_{\infty\:}\left(\%\right)={K}_{P-S\left(1\right)}\bullet\:{t}^{n}+\:{K}_{P-S\left(2\right)}\bullet\:{t}^{2n}$

$\:{Q}_{t}/{Q}_{\infty\:}$: fraction of drug released; K_0_, K_1_, K_H_: release rate constants for zero-order, first-order and Higuchi release kinetics, respectively; K_K−P_ and K_P−S(1)_: diffusion constants, K_P−S(2)_: relaxation constant; n: exponent that characterizes the diffusion process

### In vitro cytotoxicity of formulations

Immortalized human keratinocytes (HaCaT, ATCC, Manassas, VA, USA) were cultured as monolayers in 75 cm² flasks using Dulbecco’s Modified Eagle’s Medium (DMEM) with low glucose (1 g/L), sodium pyruvate and GlutaMAX, supplemented with 10% fetal bovine serum (FBS), 1% penicillin-streptomycin (10,000 U/mL penicillin, 10,000 µg/mL streptomycin) and 0.1% fungizone. Cells were maintained under standard incubation conditions of 37 °C, 5% CO₂, and saturated humidity.

For experimental procedures, cells were seeded into 96-well plates at a density of 7.5 × 10^3^ cells per well and allowed to adhere for 24 h. Subsequently, cells were exposed to VCZ, either in aqueous dispersion or encapsulated within hyalurosomes. Additionally, vehicle controls consisting of empty hyalurosomes were also assayed to confirm their absence of cytotoxicity. The VCZ-based and empty formulations were diluted in cell culture medium to obtain final concentrations of 0.5, 5, 50, and 500 ng/mL. These concentrations were selected to mimic potential in vivo dilution scenarios, with 500 ng/mL considered a likely maximum concentration of VCZ capable of reaching deeper skin layers [17]. After 48 h of incubation with the treatments, the culture medium was replaced with an MTT solution (0.5 mg/mL in phosphate-buffered saline, PBS). Following a 3-hour incubation period, the MTT solution was removed and the resulting formazan crystals were dissolved using DMSO. Absorbance was then measured at 570 nm using a Multiskan EX microplate reader (Thermo Scientific, Waltham, MA, USA). All experiments were performed in six independent runs, each conducted in triplicate. Cell viability results are expressed as percentages relative to untreated control cells (set at 100%).

### In vitro skin permeation

The transdermal permeation of VCZ, either in aqueous dispersion or encapsulated in hyalurosomes, was investigated using vertical Franz diffusion cells (Vidrafoc, Barcelona, Spain), featuring an effective diffusion area of 0.785 cm² and a receptor chamber volume of approximately 6 mL. Donor compartments were filled with 500 µL of VCZ-loaded formulations at a concentration of 0.5 mg/mL, while the receptor compartments were filled with 0.9% sodium chloride solution. Dermatomed porcine ear skin, with a uniform thickness of 600 μm, was positioned between the donor and receptor chambers. The skin samples were obtained from pig ears supplied by the Faculty of Medicine at the University of Valencia (Valencia, Spain), collected post-mortem from animals previously used in unrelated research protocols. The assembled diffusion cells were maintained in a thermostatic water bath set at 37 ± 1 °C, ensuring that the skin surface remained at a physiological temperature of 32 ± 1 °C, with continuous magnetic stirring throughout the experiment. After a 10-hour permeation period, the amount of VCZ present in the donor as well as receptor compartments was quantified using an HPLC. Prior to skin extraction, the surface was washed with 0.5 mL of AcN/H₂O (50:50) to remove any formulation remaining on the surface that had not been absorbed. Subsequently, cryostat sectioning of the treated skin samples was performed (Leica CM1950) to obtain slices being 10, 40, 100 and 450 μm thickness, simulating stratum corneum, epidermis, superficial dermis and deep dermis, respectively. The drug content within the skin layers was then extracted with AcN and analyzed by HPLC.

### Microbial strains

The antifungal activity of VCZ both in aqueous dispersion and formulated as hyalurosomes was tested against *Candida albicans* (CECT 1394). Cultures were kept for 24 h at 36 ± 1 °C. After 24 h of incubation, the fungal suspensions were diluted with PBS in order to obtain an adequate density expressed as colony forming units per milliliter (CFU/ml).

### In vitro antifungal activity

The procedure followed to carry out this type of test was described previously by our research group [14]. Briefly, vials with 500 µl of a 1/5 diluted VCZ-based formulation, or vehicle controls (empty hyalurosomes), and 500 µl of inoculum containing 1–5 × 10^5^ CFU/mL were used. Thus, the concentration of VCZ in the diluted loaded formulations was 100 µg/mL and the final concentration in the assay vials was 50 µg/mL. The assays also included a positive control composed of an aqueous dispersion of VCZ (50 µg/mL), whose inhibitory effect was used as the reference for comparison with the different formulations, and a negative control containing 500 µl of water. All vials were incubated at 36 ± 1 °C, taking samples from each vial at 0, 6, 24 and 48 h. To collect them, 50 µL of each were diluted in 5 ml of PBS and serial decimal dilutions were subsequently prepared and seeded in Petri dishes with SDA. Following incubation at 36 ± 1 °C for 48 h, CFU were enumerated. The total *C. albicans* burden in each sample was calculated based on plates exhibiting 30 to 300 colonies, with appropriate adjustments for dilution factors and plated volumes.

### In vivo antifungal activity

Protocols for the in vivo studies using mice, were approved by the Animal Care Committee of the Faculty of Pharmacy at the University of Valencia (Spain) [reference: 2024-VSC-PEA-0081]. Male 5–6 weeks old ICR (CD-1) mice, weighing 30–35 g, (Envigo, Barcelona, Spain), were obtained from the animal facility of the Faculty of Pharmacy at the University of Valencia and were kept in a clean room at a temperature of 23 ± 1 °C, a relative humidity of 60% and a light/dark cycle of 12 h. Mice were fed a standard laboratory diet and had access to water ad libitum.

**Fig. 1:**
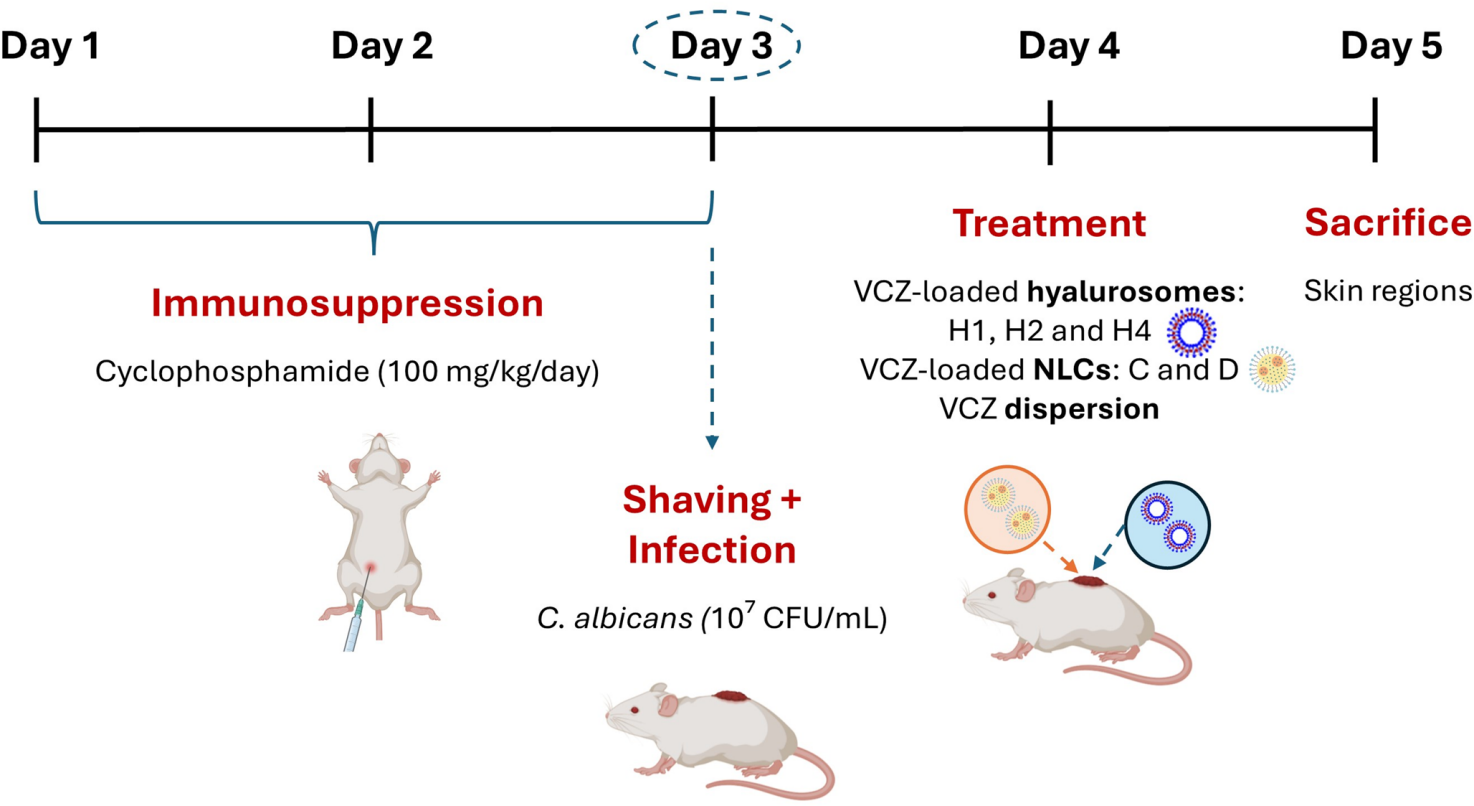
Schematic illustration of the murine in vivo antifungal activity assay

The methodology employed for these in vivo studies was based on a procedure previously published by our research team [23] and is summarized in Fig. 1. Briefly, to induce immunosuppression prior to fungal infection, mice received intraperitoneal injections of cyclophosphamide at a dose of 100 mg/kg/day for three consecutive days. On the final day of immunosuppressive treatment, a working culture of *C. albicans*, grown for 24 h at 35 °C on SDA, was used to prepare a yeast suspension containing 10⁷ CFU/mL in a mixture of RPMI 1640 and yeast extract-peptone-dextrose (YPD) medium (50/50, v/v). On the same day, the dorsal area of each mouse was shaved using an electric clipper. A 100 µL aliquot of the *C. albicans* suspension was then applied to the shaved area using a custom-designed cylindrical plastic chamber (4.5 mm i.d. × 6 mm height), which was affixed to the skin with cyanoacrylate adhesive to maintain localized contact of the suspension with the skin surface under aerobic conditions. 24 h post-inoculation, the infected skin areas were treated with 100 µL of one of the following formulations: normal saline (control), VCZ aqueous dispersion or VCZ-loaded hyalurosomes. Additionally, in order to compare the influence of the nanocarrier system in the antifungal activity of VCZ, the most promising VCZ-loaded NLCs previously developed by our research group in a previous work (formulations C and D) were evaluated [13]. All animals were sacrificed after 24 h and the corresponding skin regions were excised. The surface of each skin sample was scraped and transferred to 1 mL of Eugon LT100 broth in microcentrifuge tubes. Samples were vortexed for 30 s and centrifuged at 2,000 × g for 5 min. The supernatant was discarded and the pellet was resuspended in 1 mL of fresh Eugon LT100 broth. This wash step was repeated once. The resulting pellet was finally resuspended in 1 mL of Eugon LT100 broth and serial tenfold dilutions were prepared using the same medium. An adequate volume of sample was seeded in Petri dishes with SDA, plates were incubated at 36 ± 1 °C for 48 h and the colonies observed were counted.

### Statistical analysis

Data are presented as mean ± standard deviation (SD). The Student’s t test was used for two-group comparison. One-way analysis of variance (ANOVA) was used for comparisons of more than two groups; when statistically significant differences were found, Tukey’s test was applied to determine which groups were statistically different. *P* values of < 0.05 were considered statistically significant. All calculations were performed with IBM SPSS Statistics 26 (SPSS Inc., Chicago, IL). The values of the release kinetic parameters (estimated value ± standard error) corresponding to the different assayed models were obtained by fitting the models to the mean VCZ released (%) versus time values (Sigmaplot 10.0^®^, Systat Software, Inc., San Jose, CA, USA). Model selection was based on the coefficient of determination (R^2^), Sum of Squared Residuals (SSR) and Akaike Information Criterion (AIC) values.

## Results

### Characterization of hyalurosomes

**Fig. 2:**
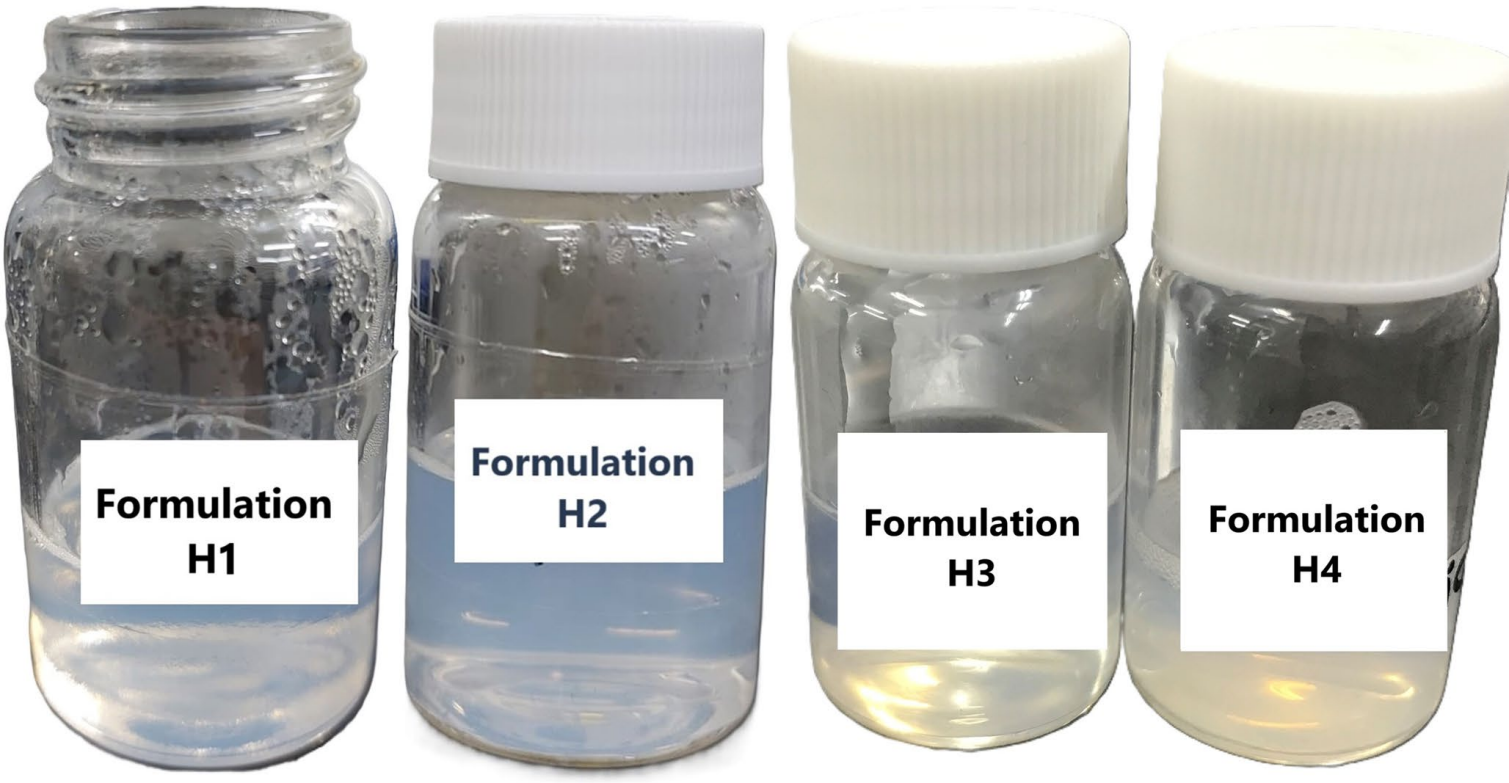
Macroscopic appearance of VCZ-loaded hyalurosomes

**Fig. 3:**
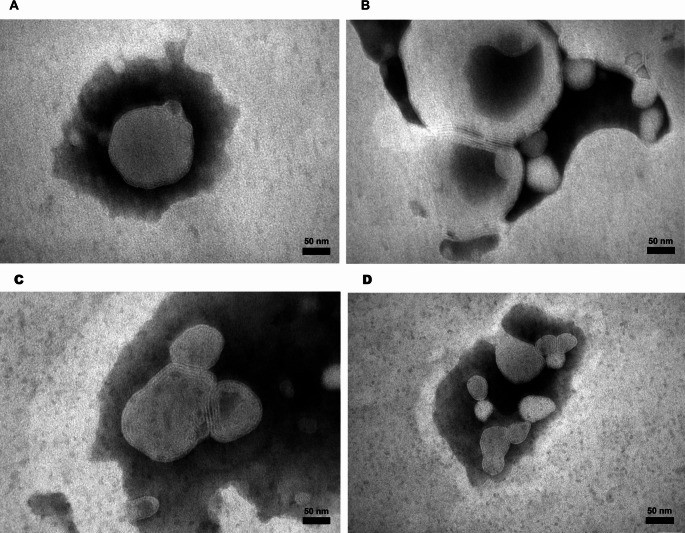
Transmission electron microscopy (TEM) images of empty formulation H3 (**A** and **B**) and VCZ-loaded formulation H3 (**C** and **D**)

The macroscopical aspect of VCZ-loaded formulations is shown in Fig. 2. The hyalurosomes were mainly multilamellar, as detected by TEM analyses. They were small in size, spherical shape and slightly aggregated (Fig. 3C and D). Empty formulations were also prepared in order to assess the effect of VCZ on hyalurosomes assembly (Fig. 3A and B).

The physicochemical properties of hyalurosomes were evaluated measuring the mean diameter (MD), polydispersity index (PI), zeta potential (ZP) and entrapment efficiency (EE) (Table 3). Nanovesicles with sizes ranging from 64 to 160 nm and PI values between 0.19 and 0.42 were obtained by varying the concentrations of phospholipid and co-solvents. Formulation H1, prepared with the lowest phospholipid concentration (1% Phospholipon 90G) and an ethanol/water mixture (10/90, v/v), produced particles with the highest MD (142 nm), and the lowest PI (0.19) and EE (72%). Furthermore, it was observed that increasing the phospholipid concentration led to a reduction in vesicle size and a slight increase in PI. EE exceeded 94% when phospholipid content was above 4%. Except for formulation H1 which was slightly negative (-12.5 mV), all formulated hyalurosomes exhibited a neutral surface charge, ranging between − 10 and + 10 mV [24].

**Fig. 4:**
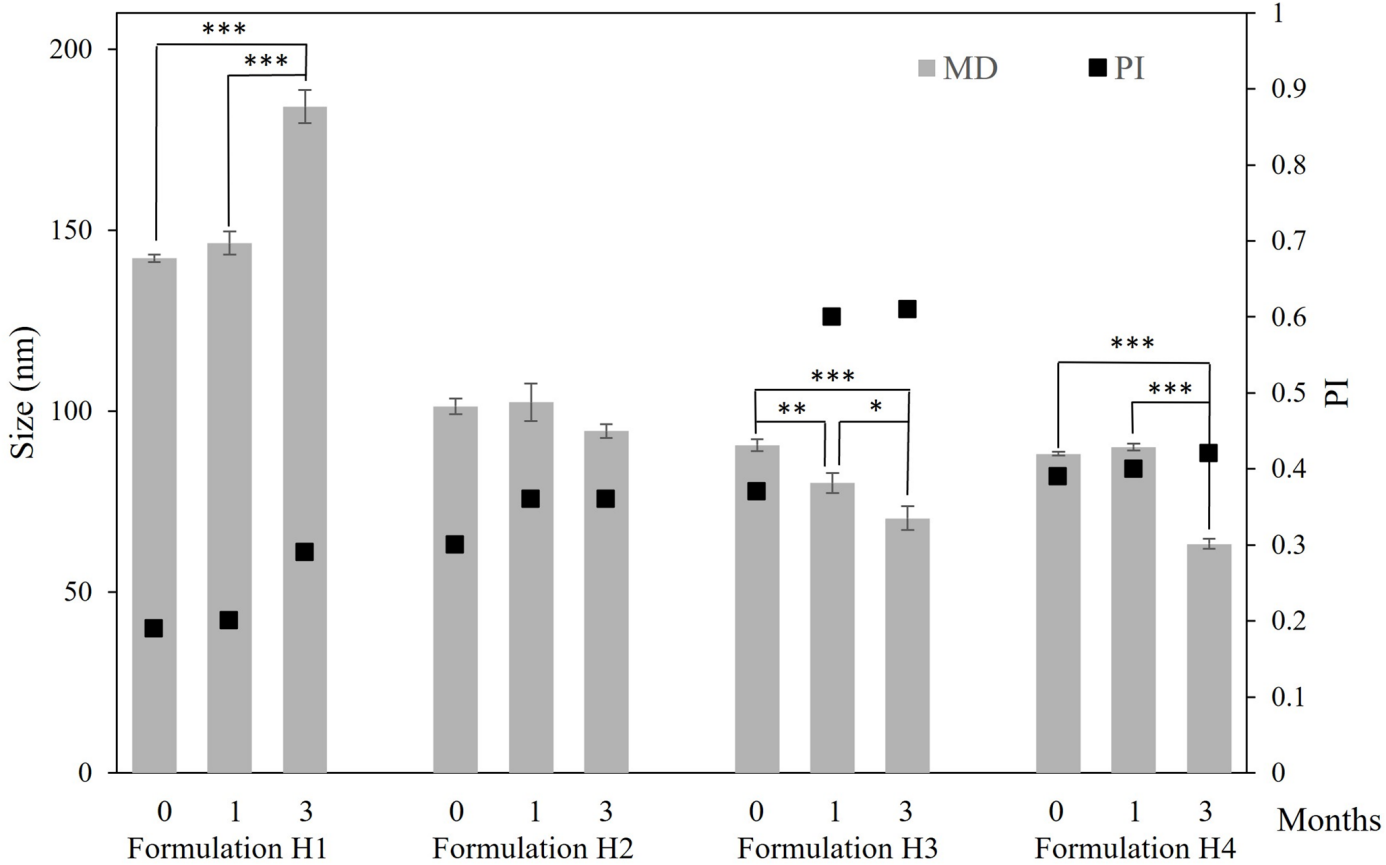
Mean diameter (MD) and polydispersity index (PI) of VCZ loaded hyalurosomes stored for 3 months at 4 °C. Data are reported as mean values ± standard deviations (SD) (*n* = 3). *p < 0.05, **p < 0.01, ***p < 0.001

**Fig. 5:**
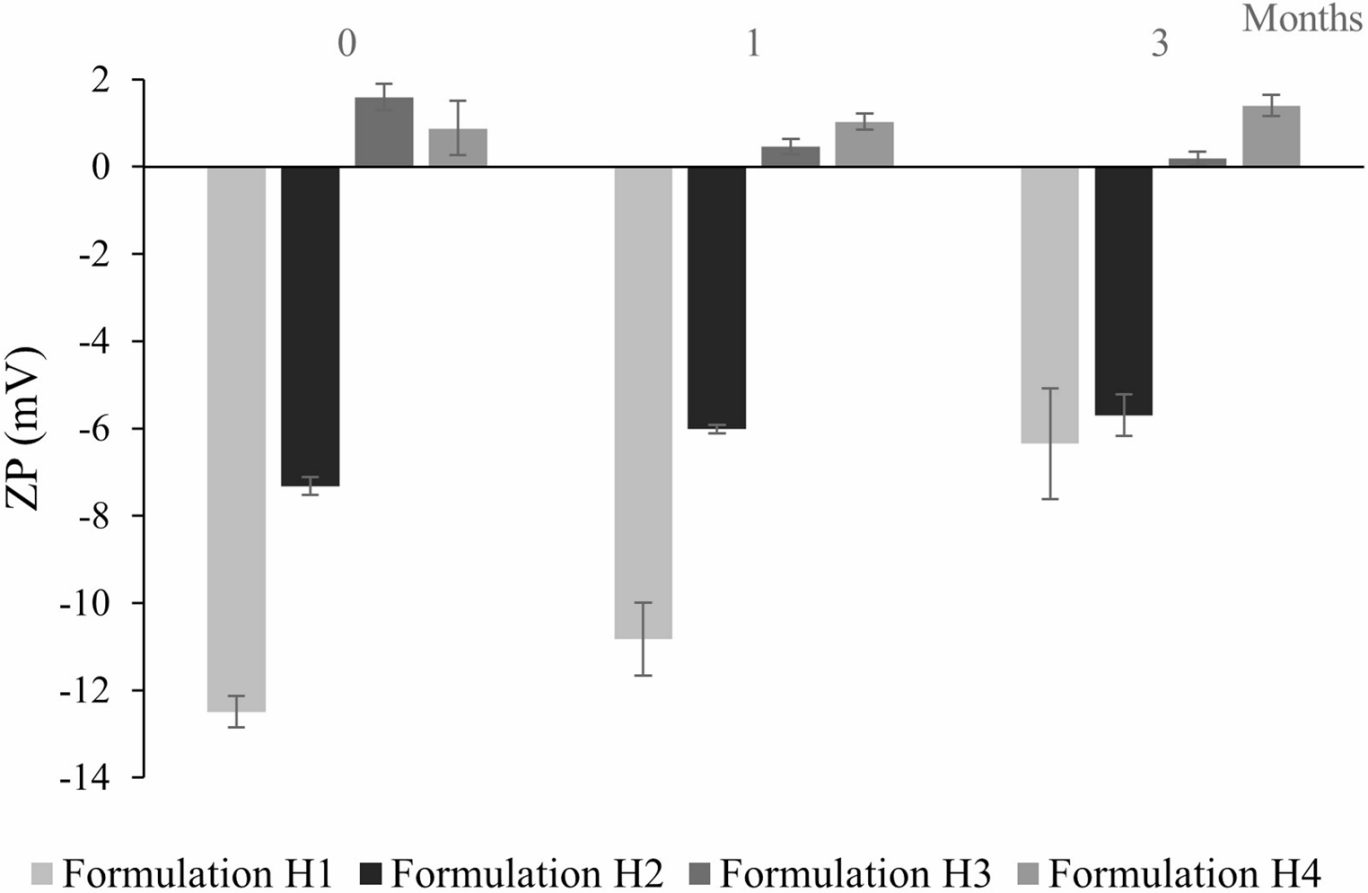
Zeta potential (ZP) of VCZ loaded hyalurosomes stored for 3 months at 4 °C. Data are reported as mean values ± SD (*n* = 3)

After 1 month of storage at 4 °C, except in formulation H3, particle size remained stable with a variation of less than 4 nm and PI below 0.4 (Fig. 4). Similarly, the ZP remained unchanged, in the neutral range (Fig. 5). The only formulation that remained stable after 3 months of storage was H2.

### In vitro release studies

**Fig. 6:**
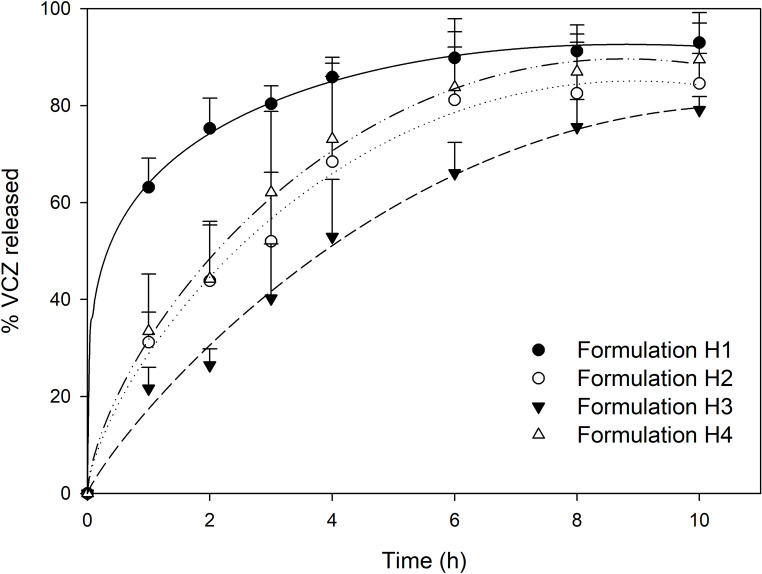
Release profiles of VCZ from the different formulations over 10 h (mean + S.D., *n* = 4). The curves show the predicted values using the Peppas-Sahlin equation

The percentages of VCZ released over time from the tested formulations are shown in Fig. 6. Between 85 and 93% of VCZ was released from hyalurosomes within 10 h. Among the formulations, H3, which contained the highest phospholipid concentration (10%), exhibited the slowest release, with only 40% of VCZ released after 3 h. In contrast, formulation H1, containing the lowest phospholipid concentration (1%), released approximately twice that amount in the same period. Formulations H2 and H4, both containing 4% of phospholipid, demonstrated intermediate release rates, suggesting that the different solvents used did not significantly influence the release of the active compound.

To describe the release kinetics, zero-order, first-order, Higuchi, Kosmeyer–Peppas and Peppas–Shalin models were tested and compared (Table 2). The kinetic models were fitted to release profiles constructed from the mean values of four independent replicates per formulation at each time point (Fig. 6). Among the applied models, the Peppas-Shalin model exhibited the best fit with the experimental data, yielding the highest coefficient of determination and the lowest AIC values. Although the first-order model provided a better statistical fit in the case of H3 (lower AIC), its simplicity limits its ability to accurately describe the physicochemical processes underlying drug release. Therefore, the Peppas–Sahlin model, which offers a more detailed interpretation of the release mechanism, was considered more appropriate for characterizing the overall behavior of the system. As can be observed in Table 4, the *n* values obtained were equal or higher than 0.5 in the case of formulations H2, H3 and H4. Therefore, in this formulations, the non-Fick or anomalous diffusion (0.5 < *n* < 1) process was dominant [25]. Moreover, as can be seen from the results, the relaxation rate constants (K_P−S(2)_) have much lower values than the diffusion rate constants (K_P−S(1)_), suggesting that the matrix nature has a relative importance compared to the Fick diffusion. In contrast, formulation H1 gave rise to an *n* value of 0.22, suggesting a Fickian release.

**Table 3:** Mean diameter (MD), polydispersity index (PI), zeta potential (ZP) and entrapment efficiency (EE) of empty and VCZ loaded hyalurosomes. Each value represents the mean value ± standard deviation (SD) of three replicates (*n* = 3)

	MD (nm)	PI	ZP (mV)	EE (%)
Empty Formulation H1	160.1 ± 3.0^a^	0.21	− 9.70 ± 0.60	-
Formulation H1	142.2 ± 1.0^b^	0.19	− 12.50 ± 0.36	71.83 ± 6.74^a^
Empty Formulation H2	78.9 ± 1.1^c^	0.25	− 5.06 ± 0.31	-
Formulation H2	101.3 ± 2.2^d^	0.30	− 7.32 ± 0.20	94.54 ± 2.41^b^
Empty Formulation H3	64.1 ± 0.5^e^	0.42	− 3.86 ± 0.12	-
Formulation H3	90.6 ± 1.7^f^	0.37	1.60 ± 0.30	94.16 ± 0.64^b^
Empty Formulation H4	91.6 ± 0.9^f^	0.39	1.81 ± 0.19	-
Formulation H4	88.2 ± 0.5^f^	0.39	0.88 ± 0.62	96.94 ± 1.36^b^

The same superscript letter indicates values that are not statistically different (*p* > 0.05)

### Biocompatibility of formulations

**Fig. 7:**
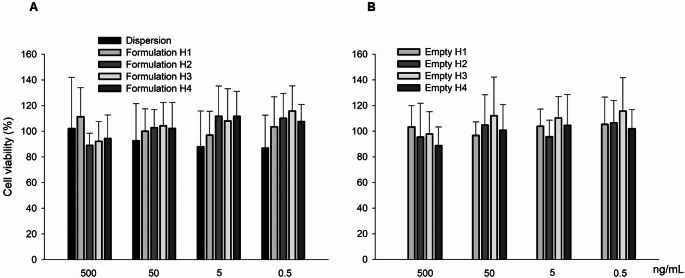
Viability of HaCaT cells after 48 h exposure to: (**A**) VCZ in dispersion or loaded in hyalurosomes, diluted to reach 500, 50, 5 or 0.5 ng/ml of VCZ; and (**B**) empty hyalurosomes. Data are reported as mean values (*n* = 17) ± SD of cell viability expressed as the percentage of untreated cells (100% of viability)

The viability of human keratinocytes (Fig. 7A) incubated for 48 h with VCZ, either in dispersion or encapsulated in hyalurosomes, was generally higher than 87% at all tested concentrations (0.5, 5, 50, and 500 ng/mL), relative to untreated cells, considered 100% viable. These findings confirm the high biocompatibility of the formulations, in line with established international standards [26]. Moreover, the results indicate that variations in the vehicle used to prepare the hyalurosomes did not affect their biocompatibility. Consistently, assays performed with the empty formulations showed cell viabilities above 89% (Fig. 7B).

### In vitro skin permeation

Percentages of VCZ present in the remaining formulation in the washing liquid, in the donor compartment, in the skin and accumulated in the receptor compartment at the end of the transdermal absorption study are summarized in Table 5. No statistically significant differences between formulations and dispersion were obtained when the percentage of VCZ in the washing liquid was compared. However, the penetration of formulation H1 and dispersion through the skin to the receptor compartment were significantly higher than those of formulations H2, H3 and H4, with higher drug percentages detected after the 10-hour penetration period. When the VCZ load on the skin was compared, statistically significant differences were obtained when formulations H1 and H3 were compared, showing a higher VCZ accumulation in the case of formulation H1.

**Table 4:** Parameters and statistical values obtained from those adjusted using the equations from release kinetics for the models used. Estimated parameter ± Standard error (SE)

Model	Parameter		Formulation			
			H1	H2	H3	H4
Zero order	K_0_ (%·h^− 1^)		13.03 ± 2.43	11.05 ± 1.31	9.56 ± 0.75	11.71 ± 1.43
	R^2^		ND	0.5585	0.8382	0.5192
	SSR		9473.18	2747.37	896.26	3308.95
	AIC		58.61	48.71	39.75	50.20
First order	K_1_ (h^− 1^)		0.71 ± 0.10	0.27 ± 0.02	0.18 ± 0.01	0.31 ± 0.02
	R^2^		0.9286	0.9696	0.9889	0.9833
	SSR		475.83	189.04	61.45	114.75
	AIC		34.68	27.30	18.31	23.31
Higuchi	K_H_ (%·h^− 1/2^)		36.85 ± 3.16	29.99 ± 1.01	25.30 ± 1.80	31.88 ± 1.14
	R^2^		0.644	0.9607	0.9724	0.9554
	SSR		2372.21	244.59	152.71	307.16
	AIC		47.54	29.36	25.59	31.18
Korsmeyer-	n		0.16 ± 0.02	0.42 ± 0.05	0.59 ± 0.05	0.40 ± 0.05
Peppas	K_K−P_ (%·h^− n^)		66.59 ± 1.70	34.69 ± 3.20	21.52 ± 2.10	37.91 ± 3.37
	R^2^		0.9948	0.9728	0.9825	0.9733
	SSR		34.35	169.25	96.72	183.50
	AIC		15.66	28.42	23.94	29.06
Peppas-Sahlin	n		0.22 ± 0.01	0.67 ± 0.08	0.86 ± 0.11	0.65 ± 0.06
	K_P−S(1)_ (%·h^− n^)		64.12 ± 0.81	29.37 ± 2.05	17.94 ± 1.74	32.28 ± 1.82
	K_P−S(2)_ (%·h^− 2n^)		~ 0	~ 0	~ 0	~ 0
	R^2^		0.9993	0.9930	0.9926	0.9951
	SSR		4.65	43.62	40.88	33.64
	AIC		1.66	19.57	19.05	17.49

ND: not determined due to poor model adjustment; K_0_, K_1_, K_H_: release rate constants for zero-order, first-order and Higuchi release kinetics, respectively; K_K−P_ and K_P−S(1)_: diffusion constants, K_P−S(2)_: relaxation constant; n: exponent that characterizes the diffusion process; R^2^: coefficient of determination; SSR: Sum of Squared Residuals; AIC: Akaike Information Criterion

**Fig. 8:**
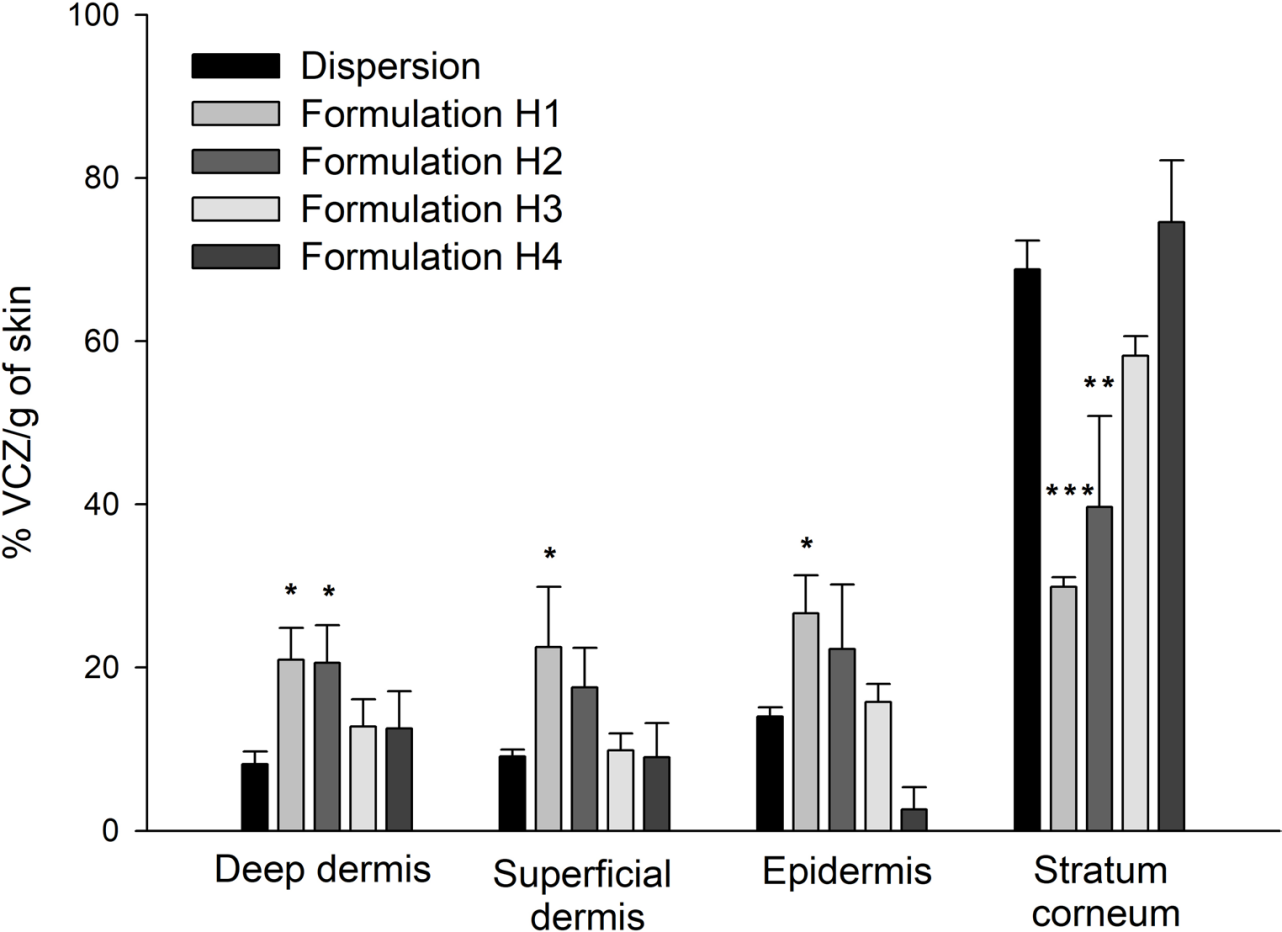
Percentage of VCZ per gram of tissue accumulated into skin layers after the administration of formulations and VCZ dispersion, following a 10-hour penetration period. Data are reported as mean values (*n* = 4) + SD. Symbols indicate that the % VCZ/g of skin treated with formulations is statistically different from that of skin treated with the dispersion. *p < 0.05, **p < 0.01, ***p < 0.001

The study of the drug distribution across skin layers revealed differences between the dispersion and formulations H1 and H2 (Fig. 8). In these formulations, VCZ was distributed more homogeneously, reaching the deepest layers of the dermis. This highlights a greater drug load in both the epidermis and dermis, which may represent a more effective reservoir for the treatment of more invasive fungal skin diseases.

### Antifungal activity on C. albicans growth

**Fig. 9:**
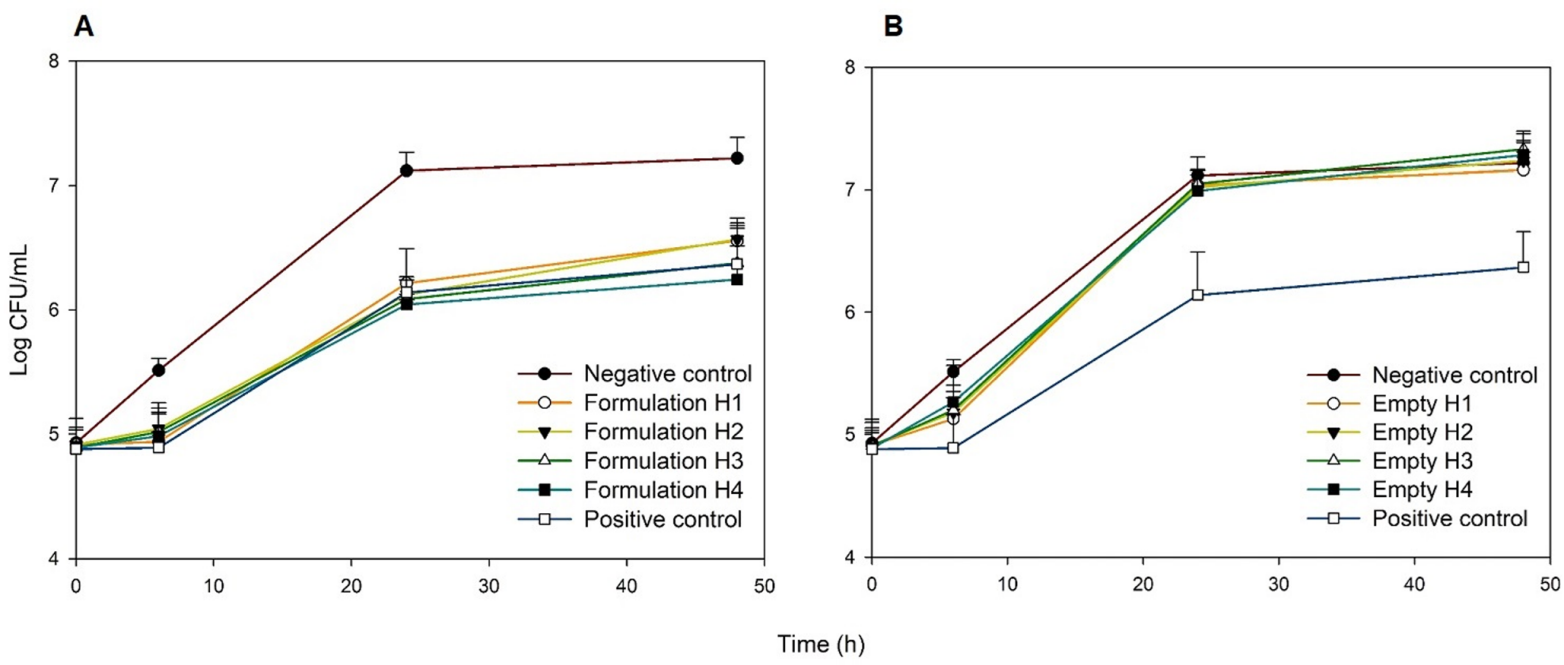
Growth curves of *C. albicans* obtained in the presence of different formulations with (**A**) or without VCZ (**B**). The curves corresponding to the positive (VCZ 50 µg/mL) and negative (without VCZ) controls are also shown for comparative purposes. Except in the negative control, the VCZ concentration in the incubation medium was 50 µg/mL. Mean + SD, *n* = 6

The growth curves of *C. albicans* in the presence of different VCZ formulations are shown in Fig. 9A. The final concentration of all formulations in the incubation medium was 50 µg/ml. In this assay, a positive control composed of an aqueous dispersion of VCZ was also tested and it was checked that it provoked the maximum inhibition of the growth. By contrast, negative control marked the absence of inhibition. As can be observed, the growth inhibition achieved by the formulations and the dispersion was similar, with a reduction of approximately 1 log after 24 h of incubation. Moreover, empty formulations were tested to confirm the absence of antifungal activity from the vehicle; the results are shown in Fig. 9B.

### In vivo antifungal activity

The antifungal activity of VCZ-loaded hyalurosomes was evaluated in vivo using mice carrying a dense layer of *C. albicans* on their dorsal skins. According to the guidelines of the ethical committee and aiming at reducing the number of used animals, only the three most promising formulations were tested, being these formulations H1, H2 and H4. The same criterion was used in the case of VCZ-loaded NLCs, only formulations C and D were tested. Saline, used in the control group, did not inhibit *C. albicans* growth.

**Fig. 10:**
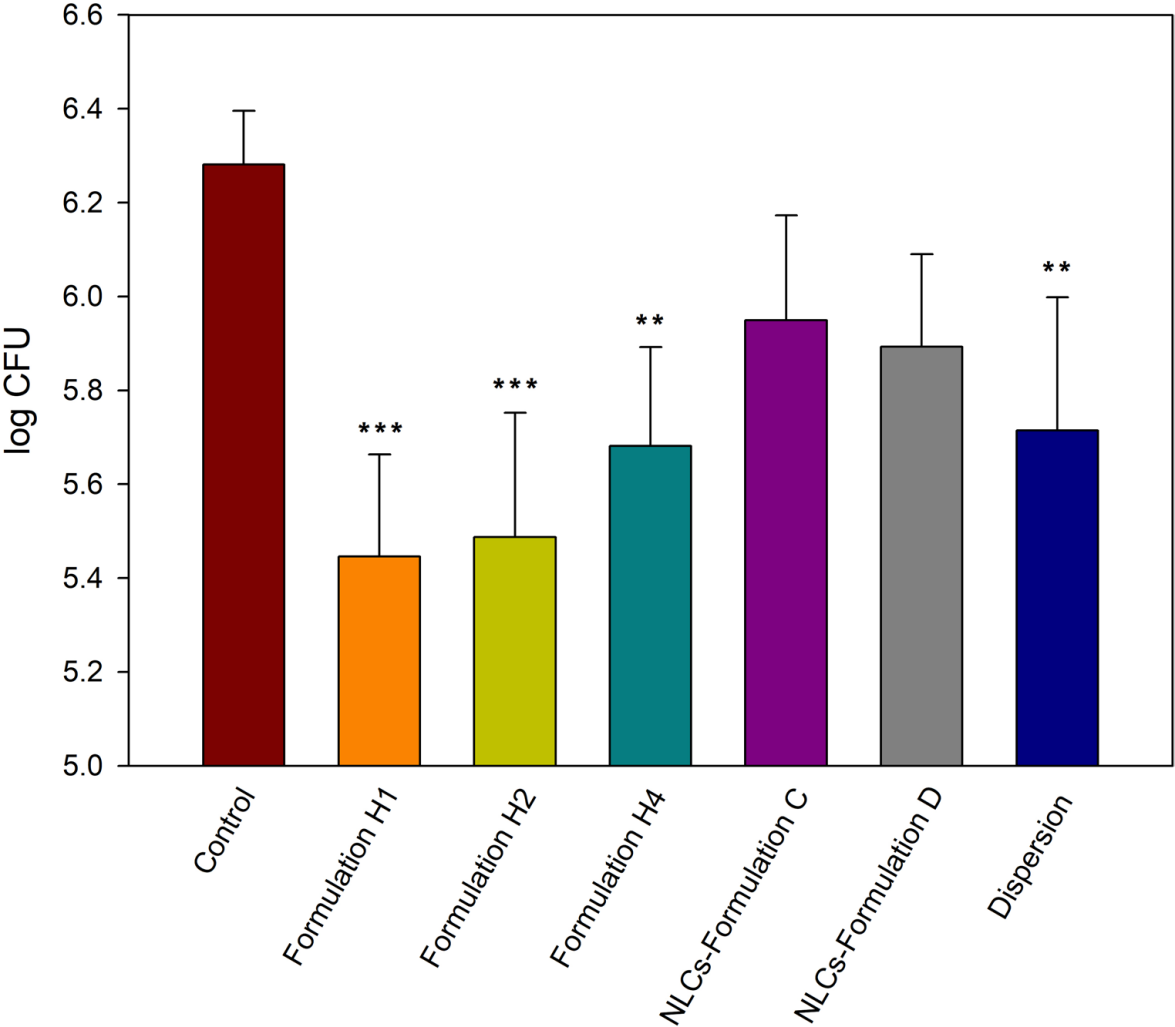
Total count of *C. albicans* (CFU) in the skin samples of mice 24 h after the administration of formulations H1, H2, H4 and VCZ dispersion. Moreover, results obtained in the case of administration of NLCs have been included (formulations C and D). Symbols indicate statistically different values from control group (saline). Mean + SD, *n* = 5. **p < 0.01, ***p < 0.001

Both the VCZ dispersion and the VCZ-loaded formulations reduced the count of the colonies of *C. albicans* (CFU) compared to the control group (Fig. 10). However, the reduction was significantly greater (*p* < 0.05) when VCZ was administered as a dispersion or formulated in hyalurosomes than when it was loaded into NLCs.

## Discussion

The development of lipid-based delivery systems loaded with VCZ has garnered significant attention in recent years, primarily to address the drug’s poor aqueous solubility, systemic side effects, and the need for targeted, sustained antifungal action. Among the most prominent VCZ-loaded nanosystems developed for topical, ocular or transungual administration are Solid Lipid Nanoparticles (SLNs) [27, 28], NLCs [13, 29–33] and different types of liposomes [34–40]. In general, they have shown an encouraging performance with regard to particle size, entrapment efficiency and in vitro release. Specifically, VCZ-loaded liposomes seem to offer the most promising features to promote deeper skin penetration, a critical factor for enhancing drug delivery to target sites within the dermis and epidermis [29, 35, 39]. However, the antifungal activity against *C. albicans* in cutaneous infections of any of these nanovesicles has been tested. In this sense, an important contribution of the present study is the in vitro and in vivo evaluation of the antifungal activity of new VCZ-loaded hyalurosomes against *C. albicans*. Indeed, unlike prior works our investigation emphasizes the impact of compositional variables on antifungal efficacy, skin penetration, and release kinetics.

The rationale behind our HA concentration (0.1%) in the four formulations developed in this work is supported by the need for a balance between hydration enhancement, skin barrier modulation, thus improving drug penetration, and the potential for synergistic wound healing and antifungal effects [17, 19, 20]. Another component present in the designed formulations that increase the flexibility of liposomes by destabilizing their lipid bilayer is the non-ionic surfactant polysorbate 80 [41], that also provides steric stabilization of colloidal systems despite a low ZP [42, 43]. One of the main difference between formulations lies in the proportion of Phospholipon^®^ 90G involved. Phospholipon^®^ 90G is a blend of purified phospholipids, derived mainly from soy lecithin. Its main component is phosphatidylcholine (≥ 94%) and may contain small proportions of other phospholipids such as phosphatidylethanolamine and phosphatidylinositol, in addition to a very low amount of triglycerides and free fatty acids. The concentration of this component can influence several characteristics of the resulting hyalurosomes, including MD, ZP, EE and drug release behavior. In this sense, an increase in Phospholipon^®^ 90G content led to a reduction in vesicle size (Table 3), a trend previously reported by Ahad et al. in the development of eprosartan mesylate-loaded transfersomes [44].

**Table 5:** VCZ percentage present in the remaining formulation in the washing liquid (% Wash), in the donor compartment (% Donor), in the skin (% Skin) and accumulated in the receptor compartment at the end of the transdermal absorption study (% Receptor). Each value represents the mean value ± SD of four replicates (*n* = 4)

	Formulation					
	Dispersion		H1	H2	H3	H4
% Wash	2.02 ± 0.71		1.22 ± 0.33	2.21 ± 0.34	1.56 ± 0.43	2.42 ± 0.34
% Donor	87.1 ± 7.1^a^		79.9 ± 3.7^a^	93.6 ± 4.6^b^	94.8 ± 2.1^b^	92.3 ± 4.1^b^
% Skin	0.32 ± 0.13^a, b^		0.51 ± 0.27^a^	0.12 ± 0.07^a, b^	0.10 ± 0.05^b^	0.13 ± 0.05ª^,b^
% Receptor	10.73 ± 7.37^a^		18.41 ± 3.05^a^	4.08 ± 2.31^b^	3.56 ± 2.11^b^	7.28 ± 2.14^b^

The same superscript letter indicates values that are not statistically different (*p* > 0.05)

With respect to ZP, the comparison between formulations H1 and H3 showed that a higher Phospholipon^®^ 90G content increased the positive surface charge, shifting the ZP toward more positive values (Table 3). This effect could be attributed to the greater abundance of choline head groups exposed on the hyalurosome surface. A similar trend was observed for formulation H4, which maintains the same phospholipid content as H2 while reducing ethanol and incorporating glycerol. The reduced ethanol content in H4 could increase the net positive charge, due to ethanol has been related to a net negative surface charge thus avoiding aggregation of vesicles due to electrostatic repulsion [45].

Similarly, EE was found to be dependent on the phospholipid concentration, with the lowest value (72%) observed at the lowest tested concentration (1% of Phospholipon^®^ 90G) (Table 3). This enhancement of EE with increasing phospholipid content is likely related to the hydrophobic nature of VCZ, which favors its incorporation into the lipid bilayer through hydrophobic interactions, thereby improving drug retention within the vesicles [39].

Despite the differences observed in size and EE between formulations, no relevant practical impact would be expected, since all obtained hyalurosomes had MD lower than 140 nm, aligning with the optimal size range for enhanced deposition in the epidermis and dermis (reported to be below 300 nm [46, 47] or around 100–150 nm [33, 48, 49], depending on the source), and high EE values (above 72%). Additionally, the slight size variations observed after at least 3 months of storage at 4 °C did not compromise the particle size suitability for topical skin administration (MD < 180 nm), considering the above reported optimal size.

The release profile analysis, based on Peppas-Sahlin kinetics, illustrates a meaningful transition from Fickian to anomalous and relaxation-controlled release as phospholipid concentration rises (Table 4). At lower phospholipid content, hyalurosomes display a greater structural disorder, the bilayer is less compact and more permeable, facilitating drug diffusion. In fact, formulation H1 (1% of Phospholipon^®^ 90G) exhibited an n-value of 0.22, indicative of a Fickian release mechanism, governed by diffusion and dependent on concentration gradient. Conversely, as the phospholipid content increases, the vesicle structure becomes more compact and stable, reducing diffusion and promoting alternative release mechanisms. This behavior was evident in formulations H2, H3, and H4. In formulations H2 and H4 (4% Phospholipon^®^ 90G), *n* = 0.6, indicating anomalous (non-Fickian) diffusion, in which both diffusion and matrix relaxation contribute to drug release. Formulation H3 (10% Phospholipon^®^ 90G) presented an n-value of 0.86, suggesting a release mechanism closer to relaxation- and/or erosion-controlled transport (anomalous or overlapping Case II), with drug release predominantly driven by structural reorganization of the lipid bilayer, swelling of hyaluronic acid, or slow disintegration of the vesicle.

Therefore, in the proposed formulations, to achieve prolonged release profiles (K_P−S(1)_ = 17.94 %·h^− n^), a higher proportion of phospholipid is recommended and, if desired to promote a faster release (K _P−S(1)_ = 64.12 %·h^− n^), a lower proportion of Phospholipon^®^ 90G is preferable. Slower, sustained release formulations are indeed advantageous, particularly for chronic conditions such as recurrent or persistent cutaneous candidiasis, where maintaining therapeutic drug levels over extended periods reduces dosing frequency and improves patient compliance. Sustained release also minimizes peak-related side effects and enhances local efficacy by ensuring constant antifungal activity at the infection site. In contrast, faster release formulations may be preferable for acute infections demanding rapid drug availability.

Consistent with the release rate, in vitro skin permeation studies revealed a significantly higher concentration of VCZ in the receptor compartment for the formulation with the fastest release (formulation H1), compared to the other formulations developed (Table 5). This effect could be attributed to the rapid saturation of the skin, allowing faster drug diffusion through the tissue. Moreover, formulation H1 demonstrated increased VCZ deposition in the deeper layers of the skin compared to the dispersion (Fig. 6), probably due to the smallest concentration of Phospholipon^®^ 90G. The inverse correlation observed in our study aligns with findings by Montenegro et al. [50], who reported that higher concentrations of this phospholipid reduce liposome flexibility and, consequently, their ability to penetrate through the stratum corneum and epidermis. However, although formulations H2 and H4 share the same phospholipid concentration, the higher ethanol content in formulation H2 (10%) enhanced VCZ penetration into deeper skin layers by disrupting stratum corneum lipids and increasing vesicle deformability [51]. In contrast, formulation H4, with less ethanol (2.5%) and more glycerol (7.5%), promoted retention in the stratum corneum. This effect can be attributed to glycerol, which improves hydration but lacks the lipid-disruptive properties needed for deeper permeation [52], thereby favoring a localized antifungal effect against superficial candidiasis while reducing systemic exposure.

Despite the differences between formulations, all of them were biocompatible with human keratinocytes and demonstrated antifungal activity against *C. albicans* comparable to that of VCZ dispersion. However, the stability of formulation H3 was the most compromised, showing loss of size homogeneity after 1 month, and significantly lower skin retention compared to formulation H1 (0.1% vs. 0.51%, respectively). Consequently, formulation H3 was excluded from in vivo testing.

In addition, in vivo assays confirmed the promising properties of formulations H1, H2 and H4, giving rise to a significant reduction in *C. albicans* growth compared to the control group (untreated). However, although VCZ-loaded NLCs previously designed by our research group exhibited favorable characteristics [13], they failed to significantly reduce *C. albicans* colony-forming units (CFU) when administered in mice. These findings highlight the superior performance of hyalurosomes over NLCs for topical antifungal therapy. The main differences between VCZ-loaded hyalurosomes and NLCs involve higher EE (mean values: 89% vs. 77%, respectively) and slower release rate (complete release at 10 vs. 6 h, respectively), and higher flexibility, which taken together favored higher penetration depth (mean value of VCZ retained in the dermis of the formulations that penetrated deeper into the skin: 41% vs. 24%, respectively) and enhanced accumulation in the receptor compartment (mean values: 8.3% vs. 1.7%, respectively). These results are consistent with those reported by Santos et al. [29], who evidenced the importance of the transfollicular route for VCZ topical delivery from liposomes and attributed the faster release of VCZ from NLCs to the drug’s lower affinity for the lipid components of NLCs compared to the phospholipids in liposomes. Additionally, the slightly hydrophilic nature of VCZ favors its retention within the internal aqueous core of liposomes [34].

## Conclusions

This study demonstrates that hyalurosomes represent a promising next-generation nanosystem for the topical delivery of VCZ, significantly enhancing antifungal activity and promoting deeper drug deposition within the skin. These results highlight the considerable potential of hyalurosomes as an advanced delivery system, effective against both superficial and invasive cutaneous candidiasis, with formulations H1 and H2 standing out as the most promising candidates for the treatment of the more invasive forms. Furthermore, by facilitating targeted local therapy and potentially reducing the risk of systemic complications, hyalurosomes address a critical need for safer and more effective antifungal interventions. Such innovation directly aligns with the World Health Organization’s global health priorities, emphasizing the urgent demand for novel solutions in the management of fungal infections by *C. albicans*.

## Data Availability

All data and materials generated or analyzed during this study are available from the corresponding author upon reasonable request.

## Data availability

The data supporting the findings of this study are included in the manuscript. Additional data are available from the corresponding author upon reasonable request.

## References

[1] Mehrmal S, Uppal P, Giesey RL, Delost GR. Identifying the prevalence and disability-adjusted life years of the most common dermatoses worldwide. J Am Acad Dermatol. 2020;82(1):258–9. 31585146 10.1016/j.jaad.2019.09.066

[2] Ziental D, Anaya-Plaza E, Talarska-Kulczyk P, Kubicka A, Żurawski J, Dlugaszewska J, et al. Quaternized phthalocyanines as a tool against melanoma and a broad spectrum of bacteria and fungi. J Photochem Photobiol B. 2025;268:113187. 40450839 10.1016/j.jphotobiol.2025.113187

[3] Eagling-Every E, Tsoi SK, Walker H, Haeusler GM. Systematic review of the Presentation, Treatment, and outcome of chronic disseminated candidiasis in children with cancer or following hematopoietic cell transplant. Pediatr Blood Cancer. 2025;72(4):e31560. 39865554 10.1002/pbc.31560

[4] World Health Organization. WHO fungal priority pathogens list to guide research, development and public health action. Geneva; 2022.

[5] Goda H, Nakashiro K, Hino S, Kuribayashi N, Uchida D. Deep cutaneous candidiasis with costal osteomyelitis following pectoralis major myocutaneous flap reconstruction: A case report. Cureus. 2025;17(1):e78210. 40027028 10.7759/cureus.78210 PMC11871034

[6] Talapko J, Juzbašić M, Matijević T, Pustijanac E, Bekić S, Kotris I, et al. Candida albicans-The virulence factors and clinical manifestations of infection. J Fungi (Basel). 2021;7(2):79. 33499276 10.3390/jof7020079 PMC7912069

[7] World Health Organization. Antifungal agents in clinical and preclinical development: overview and analysis. Geneva; 2025.

[8] Teixeira MM, Carvalho DT, Sousa E, Pinto E. New antifungal agents with Azole moieties. Pharmaceuticals (Basel). 2022;15(11):1427. 36422557 10.3390/ph15111427 PMC9698508

[9] Vitiello A, Ferrara F, Boccellino M, Ponzo A, Cimmino C, Comberiati E, et al. Antifungal Drug Resistance: Emergent Health Threat Biomedicines. 2023;11(4):1063. 37189681 10.3390/biomedicines11041063 PMC10135621

[10] Yang W, Wiederhold NP. Williams RO3. Drug delivery strategies for improved Azole antifungal action. Expert Opin Drug Deliv. 2008;5(11):1199–216. 18976131 10.1517/17425240802457188

[11] Garg A, Sharma GS, Goyal AK, Ghosh G, Si SC, Rath G. Recent advances in topical carriers of anti-fungal agents. Heliyon. 2020;6(8):e04663. 32904164 10.1016/j.heliyon.2020.e04663 PMC7452444

[12] Erdal MS, Özhan G, Mat MC, Özsoy Y, Güngör S. Colloidal nanocarriers for the enhanced cutaneous delivery of naftifine: characterization studies and in vitro and in vivo evaluations. Int J Nanomed. 2016;11:1027–37. 10.2147/IJN.S96243 PMC4798209 27042058

[13] Nácher A, Peris JE, Díez-Sales O, Taléns-Visconti R, Manca ML, Manconi M, et al. Next-Generation topical antifungal therapy: biocompatible voriconazole nanostructured lipid carriers with enhanced skin penetration for cutaneous candidiasis. J Drug Deliv Sci Technol. 2026; 115:107657.

[14] Usach I, Martínez-Álvarez P, Peris J. Topical delivery systems containing Clotrimazole for the management of candidiasis: effect of different excipients and enhanced antifungal activity of nanovesicles. Int J Pharm. 2023;644:123287. 37536641 10.1016/j.ijpharm.2023.123287

[15] Taléns-Visconti R, Perra M, Ruiz-Saurí A, Nácher A. New vehiculation systems of Mometasone furoate for the treatment of inflammatory skin diseases. Pharmaceutics. 2022;14(12):2558. 36559053 10.3390/pharmaceutics14122558 PMC9786812

[16] Pleguezuelos-Villa M, Castangia I, Diez-Sales O, Manca ML, Manconi M, Ruiz Sauri A, et al. Control of skin damages caused by oxidative stress using mangiferin and naringin co-loaded in phospholipid vesicles. J Drug Deliv Sci Technol. 2024;91:105261.

[17] Perra M, Fancello L, Castangia I, Allaw M, Escribano-Ferrer E, Peris JE, et al. Formulation and testing of antioxidant and protective effect of hyalurosomes loading extract rich in Rosmarinic acid biotechnologically produced from Lavandula angustifolia miller. Molecules. 2022;27(8):2423. 35458621 10.3390/molecules27082423 PMC9029676

[18] Lens M. Phospholipid-Based vesicular systems as carriers for the delivery of active cosmeceutical ingredients. Int J Mol Sci. 2025;26(6):2484. 40141127 10.3390/ijms26062484 PMC11942248

[19] Manca ML, Castangia I, Zaru M, Nácher A, Valenti D, Fernàndez-Busquets X, et al. Development of Curcumin loaded sodium hyaluronate immobilized vesicles (hyalurosomes) and their potential on skin inflammation and wound restoring. Biomaterials. 2015;71:100–9. 26321058 10.1016/j.biomaterials.2015.08.034

[20] Sakai A, Akifusa S, Itano N, Kimata K, Kawamura T, Koseki T, et al. Potential role of high molecular weight hyaluronan in the anti-Candida activity of human oral epithelial cells. Med Mycol. 2007;45(1):73–9. 17325947 10.1080/13693780601039607

[21] Komeil IA, Abdallah OY, El-Refaie WM. Surface modified genistein phytosome for breast cancer treatment: In-vitro appraisal, pharmacokinetics, and in-vivo antitumor efficacy. Eur J Pharm Sci. 2022;179:106297. 36156294 10.1016/j.ejps.2022.106297

[22] Usach I, Alaimo A, Fernández J, Ambrosini A, Mocini S, Ochiuz L, et al. Magnolol and honokiol: two natural compounds with similar chemical structure but different physicochemical and stability properties. Pharmaceutics. 2021;13(2):224. 33561940 10.3390/pharmaceutics13020224 PMC7915353

[23] Manca ML, Usach I, Peris JE, Ibba A, Orrù G, Valenti D, et al. Optimization of innovative Three-Dimensionally-Structured hybrid vesicles to improve the cutaneous delivery of Clotrimazole for the treatment of topical candidiasis. Pharmaceutics. 2019;11(6):263. 31174342 10.3390/pharmaceutics11060263 PMC6630241

[24] Clogston JD, Patri AK. Zeta potential measurement. Methods Mol Biol. 2011;697:63–70. 21116954 10.1007/978-1-60327-198-1_6

[25] Peppas NA. Analysis of Fickian and non-Fickian drug release from polymers. Pharm Acta Helv. 1985;60(4):110–1. 4011621

[26] ISO 10993-5. :2009 biological evaluation of medical Devices. Part 5: tests for. Vitro cytotoxicity. Geneva, Switzerland: International Organization for Standardization; 2009.

[27] Khare A, Singh I, Pawar P, Grover K. Design and evaluation of voriconazole loaded solid lipid nanoparticles for ophthalmic application. J Drug Deliv. 2016;2016:6590361. 27293896 10.1155/2016/6590361 PMC4880687

[28] Füredi P, Pápay ZE, Kovács K, Kiss BD, Ludányi K, Antal I, et al. Development and characterization of the voriconazole loaded lipid-based nanoparticles. J Pharm Biomed Anal. 2017;132:184–9. 27750101 10.1016/j.jpba.2016.09.047

[29] Santos GA, Angelo T, Andrade LM, Silva SMM, Magalhães PO, Cunha-Filho M, et al. The role of formulation and follicular pathway in voriconazole cutaneous delivery from liposomes and nanostructured lipid carriers. Colloids Surf B Biointerfaces. 2018;170:341–6. 29940500 10.1016/j.colsurfb.2018.06.037

[30] Andrade LM, Rocha KAD, De Sá, Fernando AP, Marreto RN, Lima EM, Gratieri T, et al. Voriconazole-Loaded nanostructured lipid carriers for ocular drug delivery. Cornea. 2016;35(6):866–71. 27055213 10.1097/ICO.0000000000000825

[31] Rocha KAD, Krawczyk-Santos AP, Andrade LM, Souza LCd, Marreto RN, Gratieri T, et al. Voriconazole-loaded nanostructured lipid carriers (NLC) for drug delivery in deeper regions of the nail plate. Int J Pharm. 2017;531(1):292–8. 28859937 10.1016/j.ijpharm.2017.08.115

[32] Song SH, Lee KM, Kang JB, Lee SG, Kang MJ, Choi YW. Improved skin delivery of voriconazole with a nanostructured lipid carrier-based hydrogel formulation. Chem Pharm Bull (Tokyo). 2014;62(8):793–8. 25087631 10.1248/cpb.c14-00202

[33] Waghule T, Rapalli VK, Singhvi G, Manchanda P, Hans N, Dubey SK, et al. Voriconazole loaded nanostructured lipid carriers based topical delivery system: QbD based designing, characterization, in-vitro and ex-vivo evaluation. J Drug Deliv Sci Technol. 2019;52:303–15.

[34] de Sá FA, Pires, Taveira SF, Gelfuso GM, Lima EM, Gratieri T. Liposomal voriconazole (VOR) formulation for improved ocular delivery. Colloids Surf B Biointerfaces. 2015;133:331–8. 26123854 10.1016/j.colsurfb.2015.06.036

[35] Song CK, Balakrishnan P, Shim C, Chung S, Chong S, Kim D. A novel vesicular carrier, transethosome, for enhanced skin delivery of voriconazole: characterization and in vitro/in vivo evaluation. Colloids Surf B Biointerfaces. 2012;92:299–304. 22205066 10.1016/j.colsurfb.2011.12.004

[36] Hassanpour P, Hamishehkar H, Baradaran B, Mohammadi M, Shomali N, Spotin A, et al. An appraisal of antifungal impacts of nano-liposome containing voriconazole on voriconazole-resistant Aspergillus flavus isolates as a groundbreaking drug delivery system. Nanomed Res J. 2020;5(1):90–100.

[37] Hassanpour P, Hamishehkar H, Bahari Baroughi B, Baradaran B, Sandoghchian Shotorbani S, Mohammadi M, et al. Antifungal effects of Voriconazole-Loaded Nano-Liposome on Fluconazole-Resistant clinical isolates of Candida albicans, biological activity and ERG11, CDR1, and CDR2 gene expression. Assay Drug Dev Technol. 2021;19(7):453–62. 34435891 10.1089/adt.2020.1057

[38] Alhakamy NA, Hosny KM, Rizg WY, Eshmawi BA, Badr MY, Safhi AY, et al. Development and optimization of hyaluronic Acid-Poloxamer In-Situ gel loaded with voriconazole cubosomes for enhancement of activity against ocular fungal infection. Gels. 2022;8(4):241. 35448142 10.3390/gels8040241 PMC9032757

[39] Faisal W, Soliman GM, Hamdan AM. Enhanced skin deposition and delivery of voriconazole using ethosomal preparations. J Liposome Res. 2018;28(1):14–21. 27667097 10.1080/08982104.2016.1239636

[40] Fahmy AM, Hassan M, El-Setouhy DA, Tayel SA, Al-Mahallawi AM. Statistical optimization of hyaluronic acid enriched ultradeformable elastosomes for ocular delivery of voriconazole via Box-Behnken design: in vitro characterization and in vivo evaluation. Drug Deliv. 2021;28(1):77–86. 33342315 10.1080/10717544.2020.1858997 PMC7875553

[41] El Maghraby GMM, Williams AC, Barry BW. Interactions of surfactants (edge activators) and skin penetration enhancers with liposomes. Int J Pharm. 2004;276(1–2):143–61. 15113622 10.1016/j.ijpharm.2004.02.024

[42] Yemparala V, Damre AA, Manohar V, Sharan Singh K, Mahajan GB, Sawant SN, et al. Effect of the excipient concentration on the pharmacokinetics of PM181104, a novel antimicrobial Thiazolyl Cyclic peptide antibiotic, following intravenous administration to mice. Results Pharma Sci. 2014;4:34–41. 25756005 10.1016/j.rinphs.2014.09.001 PMC4348513

[43] Mohd Izham MN, Hussin Y, Aziz MNM, Yeap SK, Rahman HS, Masarudin MJ, et al. Preparation and characterization of self Nano-Emulsifying drug delivery system loaded with Citraland its antiproliferative effect on colorectal cells in vitro. Nanomaterials (Basel). 2019;9(7):1028. 31323842 10.3390/nano9071028 PMC6669672

[44] Ahad A, Al-Saleh AA, Al-Mohizea AM, Al-Jenoobi FI, Raish M, Yassin AEB, et al. Formulation and characterization of phospholipon 90 G and tween 80 based transfersomes for transdermal delivery of Eprosartan mesylate. Pharm Dev Technol. 2018;23(8):787–93. 28504046 10.1080/10837450.2017.1330345

[45] Verma P, Pathak K. Nanosized ethanolic vesicles loaded with econazole nitrate for the treatment of deep fungal infections through topical gel formulation. Nanomedicine. 2012;8(4):489–96. 21839053 10.1016/j.nano.2011.07.004

[46] Akombaetwa N, Ilangala AB, Thom L, Memvanga PB, Witika BA, Buya AB. Current advances in lipid nanosystems intended for topical and transdermal drug delivery applications. Pharmaceutics. 2023;15(2):656. 36839978 10.3390/pharmaceutics15020656 PMC9967415

[47] Verma DD, Verma S, Blume G, Fahr A. Particle size of liposomes influences dermal delivery of substances into skin. Int J Pharm. 2003;258(1–2):141–51. 12753761 10.1016/s0378-5173(03)00183-2

[48] Santonocito D, Puglia C. Lipid nanoparticles and skin: discoveries and advances. Cosmetics. 2025;12(1):22.

[49] Mardhiah Adib Z, Ghanbarzadeh S, Kouhsoltani M, Yari Khosroshahi A, Hamishehkar H. The effect of particle size on the deposition of solid lipid nanoparticles in different skin layers: A histological study. Adv Pharm Bull. 2016;6(1):31–6. 27123415 10.15171/apb.2016.06 PMC4845546

[50] Montenegro L, Paolino D, Drago R, Pignatello R, Fresta M, Puglisi G. Influence of liposome composition on in vitro permeation of diosmin through human stratum corneum and epidermis. J Drug Deliv Sci Technol. 2006;16(2):133–40.

[51] Verma P, Pathak K. Therapeutic and cosmeceutical potential of ethosomes: an overview. J Adv Pharm Technol Res. 2010;1(3):274–82. 22247858 10.4103/0110-5558.72415 PMC3255417

[52] Hara M, Verkman AS. Glycerol replacement corrects defective skin hydration, elasticity, and barrier function in aquaporin-3-deficient mice. Proc Natl Acad Sci U S A. 2003;100(12):7360–5. 12771381 10.1073/pnas.1230416100 PMC165880

